# ﻿Morphological and molecular characterisation of the Popijač’s Yellow Sally, *Isoperlapopijaci* sp. nov., a new stenoendemic stonefly species from Croatia (Plecoptera, Perlodidae)

**DOI:** 10.3897/zookeys.1078.66382

**Published:** 2021-12-16

**Authors:** Dora Hlebec, Ignac Sivec, Martina Podnar, Josip Skejo, Mladen Kučinić

**Affiliations:** 1 Department of Biology, Faculty of Science, University of Zagreb, Rooseveltov trg 6, 10000 Zagreb, Croatia University of Zagreb Zagreb Croatia; 2 Slovenian Museum of Natural History, Prešernova 20, 1000 Ljubljana, Slovenia Slovenian Museum of Natural History Ljubljana Slovenia; 3 Croatian Natural History Museum, Demetrova 1, 10000 Zagreb, Croatia Croatian Natural History Museum Zagreb Croatia

**Keywords:** Conservation, Dinaric karst, DNA barcoding, *Isoperlapopijaci* sp. nov., karstic source, species delimitation

## Abstract

A new species of the Yellow Sally genus (*Isoperla* Banks, 1906) is described, based on morphological (males and females adults, larval and egg) and molecular (the barcode region of the cytochrome c oxidase subunit I gene (*COI*)) features. Popijač’s Yellow Sally, *I.popijaci* Hlebec & Sivec, **sp. nov.** inhabits two karstic sources of the Krasulja rivulet in Croatia. Male and female of the new species are characterised by colouration patterns of the head and pronotum; the dimensions of the female subgenital plate; the medial penial armature and oval-shaped egg without collar and anchor. The larvae differ from their congeners by the uniquely coloured head and pronotum. Based on morphological characteristics *I.popijaci***sp. nov.** belongs to the *I.tripartita* species group. Phylogenetic and taxonomic relationships were reconstructed using three methods of phylogenetic inference and three species delimitation methods. As *I.popijaci***sp. nov.** occurs at a narrow area of the Krasulja rivulet in Krbava field, the study puts emphasis on the conservation and hotspot importance of the temporary rivers in the Dinaric karst. Furthermore, the study accentuates the necessity for further research on the genetic diversity of Plecoptera in Croatia.

## ﻿Introduction

Predominantly regarded as a biological indicator of well oxygenated water in freshwater ecosystems (Illies and Schmitz 1980; Hamid and Rawi 2017; Morinière et al. 2017; DeWalt and Ower 2019; Ferreira et al. 2020), stoneflies (Plecoptera) and their absence can indicate pollution, changes in habitat conditions, habitat destruction and climate changes (Urbanič and Toman 2007; Fochetti and Tierno de Figueroa 2008; Bálint et al. 2011). In total, 50 Plecoptera species are reported from Croatia and, due to the many suitable habitats, it is assumed that this number is higher (Popijač and Sivec 2009a, 2009b). Members of the subfamily Perlodinae are, in general, vividly coloured, medium to large-sized, show high genetic diversity and are often microendemic (Zwick 1973, 2004; Li and Murányi 2015). The genus *Isoperla* Banks, 1906 is represented by 188 species worldwide and 60 species in Europe (DeWalt et al. 2020). The genus has a Holarctic and Oriental distribution (Zwick 1973; Szczytko and Stewart 1979; Sandberg and Kondratieff 2013; Szczytko and Kondratieff 2015) and represents the most diverse genus of the family Perlodidae in Europe (Graf et al. 2009, 2018). Thereby, the area of the Balkan stands out as a diversity hotspot with 21 species, of which 12 are endemic to the Peninsula and often restricted to specific habitats (Murányi 2011; Murányi et al. 2016).

Basic characteristics by which the species within the genus *Isoperla* are distinguished are penial morphology, head and pronotal pattern, egg structures and drumming signals (Despax 1936; Illies 1952, 1954, 1966; Sivec and Stark 2002; Murányi 2011; Michalik et al. 2017). In the last few years, a considerable number of new Plecoptera taxa have been described, especially from China (Li et al. 2013; Ji et al. 2014; Li and Murányi 2015; Chen et al. 2019; Cao et al. 2020), but also in Europe, like *Isoperlapesici* Murányi, 2011; *I.autumnalis* Murányi, 2011; *I.citrina* Murányi, 2011 (Murányi 2011; Murányi et al. 2016); *I.vjosae* Graf et Vitecek, 2018 (Graf et al. 2018); *I.claudiae* Graf et Konar, 2014 (Graf et al. 2014) and *I.nagyi* Murányi, Kovács et Graf, 2020 (Murányi et al. 2020).

During fieldwork research since 2004, ten *Isoperla* species were recorded in Croatia. An additional one is here described as *Isoperlapopijaci* sp. nov., which shares morphological characteristics of the penial armature with species from the *I.tripartita* species group.

The following study provides a morphological description of the new species: illustrations of the main taxonomical characters (in males, females, larvae and eggs); as well as its phylogenetic placement within the genus based on the mitochondrial cytochrome c oxidase subunit I (*COI*) barcode region as a marker. Moreover, the conservation importance of the intermittent Krasulja rivulet and its watercourse, as well as Dinaric karst (Western Balkan region) is discussed.

## ﻿Materials and methods

### ﻿Material collection and preparation.

Adults of *I.popijaci* sp. nov. were collected in June 2019 at the entrance to the Ševerova Cave (karstic source of the intermittent Krasulja rivulet in Krbava field). A subsequent collecting trip upstream of the Krasulja rivulet (in June 2021), near the karstic source adjacent to the village of Mirići, resulted in finding more specimens of *I.popijaci* sp. nov.

A total of 42 specimens (34 adults and 8 larvae) belonging to *Isoperlapopijaci* sp. nov., were collected. Adult specimens were collected using sweep nets, while larval specimens were collected by handpicking. The aedeagus was everted in the field and specimens were fixed and stored in 96% ethanol for morphological and molecular analysis. Morphological characteristics of male terminalia were examined after potassium hydroxide (KOH) treatment.

### ﻿Type material depository and museum acronyms.

The holotype and part of the paratypes series are deposited in the Croatian Natural History Museum, Zagreb, Croatia (CNHM), Collection of Plecoptera Sivec & Hlebec, while other paratypes are kept in the Slovenian Museum of Natural History, Ljubljana, Slovenia (PMSL).

### ﻿Photography and drawings.

Photographs, diagnostic characterisation and comparative morphological examination of specimens were made using a ZEISS SteREO DiscoveryV.20 stereomicroscope. Pencil drawings were produced with a camera lucida and then digitally edited and inked. Figures 3A, B, 4A–D (SEM images) were made using a JEOL JSM-7000F scanning electron microscope. The penis (one of paratype specimen) for the SEM study was critical-point dried (Figure 4A–D).

Nomenclature is in accordance with the International Code of the Zoological Nomenclature (ICZN 1999). The species is proposed by following the rules of the Code. Abbreviations for the type specimens are HT–holotype, PT–paratype and PTs–paratypes.

### ﻿Comparative analysis.

Comparative study on the morphology of penial structures was conducted using ten species belonging to the genus *Isoperla*, collected in Croatia: *I.bosnica* Aubert, 1964; *I.inermis* Kaćanski et Zwick, 1970; *I.rivulorum* (Pictet, 1841); *I.lugens* (Klapálek, 1923); *I.illyrica* Tabacaru, 1971; *I.tripartita* Illies, 1954; *I.grammatica* (Poda, 1761); *I.difformis* (Klapálek, 1909); *I.oxylepis* (Despax, 1936) and *I.albanica* Aubert, 1964. Morphological taxonomic classifications follow the traditional system (Poda 1761; Pictet 1841; Klapálek 1909, 1923; Despax 1936; Illies 1952, 1954, 1966; Aubert 1964; Tabacaru 1971; Kaćanski and Zwick 1970, Murányi 2011; Murányi et al. 2016).

### ﻿DNA extraction, amplification, and sequencing.

One male, one female and one larva of *Isoperlapopijaci* sp. nov. were used in molecular analyses and mutually associated. DNA was extracted from the single leg of specimens using QIAamp DNA Micro Kit (Qiagen, Germany) according to the manufacturer’s specifications and eluted in 50 µl of elution buffer. The 5’ fragment of the mitochondrial cytochrome c oxidase subunit I gene (*COI*) was amplified using standard PCR-protocols and four sets of primers: LCO-1490/HCO-2198 (Folmer et al. 1994) or C_LepFolF/C_LepFolR (as was used in Hebert et al. 2004) or a combination of MLepF1/LepR1 and MLepR1/LepF1 (yielding two shorter, overlapping fragments as was used in Hajibabaei et al. 2006) in 20 µl reactions. Polymerase chain reactions (PCRs) for all primer sets were carried out using: 1 x DreamTaq reaction buffer with 2 mM MgCl_2_ (Thermo Fisher Scientific Inc., US), 0.2 mM dNTPs, 0.4 µM of each primer, 0.025 U/µl of DreamTaq polymerase (Thermo Fisher Scientific Inc., US) and 1 µl of eluted DNA. For the first mentioned primers set (LCO-1490/HCO-2198) the following PCR cycling conditions were applied: initial denaturation at 95°C for 2 min, followed by 35 cycles of denaturation at 95°C for 30 s, annealing at 50°C for 30 s, extension at 72°C for 1 min, followed by a final extension step at 72°C for 10 min. PCR products were purified using Exonuclease I (0.05 U/µl), FastAP Thermosensitive Alkaline Phosphatase (0.025 U/µl) enzymatic system (Thermo Fisher Scientific Inc., US). The reaction was carried using the protocol: 1 h at 37°C followed by 20 min at 80°C. Sequencing was performed by Macrogen Inc. (Amsterdam, The Netherlands) using the amplification primers. Sequences obtained in the study were deposited in the BOLD database (Ratnasingham and Hebert 2007) and GenBank (under the accession numbers MW907977–MW907980, MW907982–MW907988 and MW907990–MW907993).

### ﻿Sequence data and phylogenetic analysis.

In total, 15 obtained *Isoperla* sequences were checked, edited, assembled from both directions and inspected manually for base-pair ambiguities, as well as stop codons, indels or double peaks in chromatograms (as indicators for the possible erroneous amplification of nuclear mitochondrial pseudogene) in Geneious R6 (https://www.geneious.com). All available *Isoperla* sequences were retrieved from the GenBank and BOLD databases (accessed 10/01/2021) and aligned with sequences from this study using MAFFT v.7 (Katoh and Standley 2013). Any length variants were excluded from the final alignments. Sequences were collapsed into 456 unique *COI* haplotypes using the online tool FaBox v.1.5 (Villesen 2007) and, from all species, the most diverse haplotypes from *I.tripartita* and *I.rivulorum* species group, as well as species *I.lugens*, were retained for further analysis. The final dataset for phylogenetic analysis and species delineation comprised 27 sequences, including 10 haplotypes observed in this study (see Table 1). *Isoperlaobscura* (INTAP055-17) and *Taeniopteryxburksi* (08INHSP-002) were selected as outgroups according to the North American Plecoptera phylogeny published by South et al. (2020). Amongst morphologically-defined species, evolutionary divergence was estimated using the pairwise comparison of the uncorrected genetic distances (*p*-distances) in MEGA-X (Kumar et al. 2018). For *p*-distances, a colour heat map was drawn using the Python data visualisation library Seaborn (version 0.11.1, Waskom 2021). Phylogenetic relationships were estimated by three different optimality criteria: Neighbour Joining (NJ), Maximum Likelihood (ML) and Bayesian Inference (BI). NJ and ML were performed in MEGA-X (Kumar et al. 2018), while BI in MrBayes 3.2.7. (Ronquist et al. 2012). For ML and BI, the optimal model of nucleotide evolution (Hasegawa-Kishino-Yano model with gamma distributed rate variation amongst sites and a significant proportion of invariable sites: HKY+I+G) was selected under the Bayesian Information Criterion (BIC) using jModelTest 2.1.5 (Darriba et al. 2012). Nodes in the phylogenetic trees with bootstrap values P ≥ 70 in NJ and ML and posterior probabilities values pp ≥ 0.90 in BI were considered well supported. NJ was made using the Kimura-2-parameter (K2P) model of nucleotide substitution with the pairwise deletion option. Bootstrap support was inferred using the fast bootstrap algorithm, based on 5000 replicates. Nearest-Neighbour-Interchange (NNI), a heuristic method using the fast bootstrap algorithm, was used in ML with 1000 replicates.

**Table 1.** Collection details and geographical origin of the specimens used in phylogenetic analysis. Haplotypes obtained in this study, marked with asterisk. Paratypes of *Isoperlapopijaci* sp. nov. used in molecular analysis marked in bold (male, female and larval). Abbreviations: AL (Albania), AT (Austria), C (Croatia), F (France), G (Germany), M (Montenegro), S (Switzerland). Outgroups (INTAP055-17 and 08INHSP-002) are not shown. Specimen identifier: I. Sivec.

Specimen ID	BOLD/GenBank Process ID	Taxon	Locality	Legit	Coordinates	Publication
*DH71	CROPL066–21	*Isoperlarivulorum*	C: Kupa River, spring	I. Sivec	45°29.47'N, 14°41.36'E	this study
GBOL01391	GBCOU1198-13	*Isoperlarivulorum*	F: Rhone-Alpes, Hauteville	Balke, Morinière, Toussaint, Taenzler, Bellanger, Hoch	45°29.52'N, 6°35.04'E	Morinière et al. (2017)
PE219	INTAP187-17	*Isoperlarivulorum*	AT: Flexenpass	W. Graf	47°09.17'N, 10°09.91'E	–
PE268	INTAP226-17	*Isoperlarivulorum*	AT: Flexenpass	W. Graf	47°09.17'N, 10°09.91'E	–
GBIFCH00280047	PLEAA237-20	*Isoperlarivulorum*	S: Effluent, Pont de Nant	Sartori Michel & Derleth Pascale	46°15.07'N, 7°06.43'E	–
GBOL01390	GBCOU1197-13	*Isoperlarivulorum*	F: Rhone-Alpes, Hauteville	Balke, Morinière, Toussaint, Taenzler, Bellanger, Hoch	45°29.52'N, 6°35.04'E	Morinière et al. (2017)
*DH107	CROPL097–21	*Isoperlaillyrica*	C: Trilj, Grab, spring	I. Sivec	43°38.93'N, 16°45.74'E	this study
*DH482	CROPL197–21	*Isoperlaillyrica*	C: Trilj, Grab, spring	I. Sivec	43°38.93'N, 16°45.74'E	this study
*DH123	CROPL109–21	*Isoperlatripartita*	C: Cetina River, spring	I. Sivec	43°58.54'N, 16°25.81'E	this study
*DH478	CROPL195–21	*Isoperlatripartita*	C: Cetina River, spring	B. Horvat	43°58.54'N, 16°25.81'E	this study
*DH551	CROPL225–21	*Isoperlatripartita*	C: Papuk, Gospin potok	I. Vučković	45°34.47'N, 17°41.76'E	this study
*DH137	CROPL122–21	*Isoperlatripartita*	C: Trilj, Grab, spring	I. Sivec	43°38.93'N, 16°45.74'E	this study
Itri0101M	VJOSA001-17	*Isoperlatripartita*	AT: Lainzer Tiergarten	O. Zweidick	48°09.57'N, 16°12.83'E	Graf et al. (2018)
Itri0102M	VJOSA002-17	*Isoperlatripartita*	AT: Lainzer Tiergarten	O. Zweidick	48°09.57'N, 16°12.83'E	Graf et al. (2018)
MT348738	GBMNC47893-20	*Isoperlatripartita*	Macedonia	D. Murányi	41°16.07'N, 20°31.24'E	Murányi et al. (2020)
MT348735	GBMNC47896-20	*Isoperlatripartita*	Macedonia	D. Murányi	42°03.14'N, 20°46.92'E	Murányi et al. (2020)
MT348732	GBMNC47899-20	*Isoperlatripartita*	Macedonia	D. Murányi	40°58.78'N, 21°15.22'E	Murányi et al. (2020)
**DH129**	CROPL115–21	*Isoperlapopijaci* sp. nov.	C: Ševerova Cave	I. Sivec	44°40.78'N, 15°37.87'E	this study
**DH130**	CROPL116–21	*Isoperlapopijaci* sp. nov.	C: Ševerova Cave	I. Sivec	44°40.78'N, 15°37.87'E	this study
*DH926	CROPL249–21	*Isoperlapopijaci* sp. nov.	C: Ševerova Cave	D. Hlebec	44°40.78'N, 15°37.87'E	this study
DH142	CROPL127–21	*Isoperla* PL	C: Plitvice Lakes, Drakulića River	I. Sivec	44°46.87'N, 15°39.47'E	this study
DH143	CROPL128–21	*Isoperla* PL	C: Plitvice Lakes, Drakulića River	I. Sivec	44°46.87'N, 15°39.47'E	this study
*DH538	CROPL214–21	*Isoperla* PL	C: Plitvice Lakes, Drakulića River	M. Kučinić, I. Vučković	44°46.87'N, 15°39.47'E	this study
DH541	CROPL217–21	*Isoperla* PL	C: Plitvice Lakes, Drakulića River	M. Kučinić, I. Vučković	44°46.87'N, 15°39.47'E	this study
*DH629	CROPL230–21	*Isoperla* PL	C: Plitvice Lakes, Drakulića River	M. Kučinić, I. Vučković	44°46.87'N, 15°39.47'E	this study
Itri0201M	VJOSA003-17	*Isoperlavjosae*	AL: Vjosa River, Kutë	S. Vitecek, W. Graf	40°28.35'N, 19°44.94'E	Graf et al. (2018)
Itri0202M	VJOSA004-17	*Isoperlavjosae*	AL: Vjosa River, Kutë	S. Vitecek, W. Graf	40°28.35'N, 19°44.94'E	Graf et al. (2018)
Itri0301M	VJOSA005-17	*Isoperlavjosae*	AL: Vjosa River, Kutë	S. Vitecek, W. Graf	–	–
Itri0302L	VJOSA006-17	*Isoperlavjosae*	AL: Vjosa River, Kutë	S. Vitecek, W. Graf	40°28.35'N, 19°44.94'E	Graf et al. (2018)
Ipe0101M	VJOSA007-17	*Isoperlapesici*	M: Redice	W. Graf	42°53.02'N, 19°18.95'E	Graf et al. (2018)
Ipe0102F	VJOSA008-17	*Isoperlapesici*	M: Redice	W. Graf	42°53.02'N, 19°18.95'E	Graf et al. (2018)
GBOL17507	GBMIX2517-15	*Isoperlalugens*	G: Nationalpark Berchtesgaden	R. Gerecke	47°33.48'N, 12°48.24'E	Morinière et al. (2017)
PE031	INTAP025-17	*Isoperlalugens*	AT: Koerbersee	W. Graf	47°16.09'N, 10°07.66'E	–
PE269	INTAP227-17	*Isoperlalugens*	AT: Flexenpass	W. Graf	47°09.17'N, 10°09.91'E	–

For BI, the dataset was partitioned by codon positions. Two separate runs with four Metropolis-coupled Monte Carlo Markov chains (MMCM) were performed for 10 million generations while trees were sampled every 1000 generations with the first 25% of sampled trees discarded as burn-in. The remaining trees were used to create a 50% majority rule consensus tree. TRACER v.1.7.1 (Rambaut et al. 2018) was used to check the convergence between the two runs. The phylogenetic trees were visualised using FigTree v.1.4.3. (Rambaut 2009) and iTOL v.5 (Letunic and Bork 2021). Several methods of species delimitation were applied: the Automatic Barcode Gap Discovery (ABGD) method (Puillandre et al. 2012), the Bayesian implementation of the Poisson Tree Processes (bPTP) method (Zhang et al. 2013) and the multi-rate Poisson Tree Process (mPTP) method (Kapli et al. 2017). The ABGD was performed at the web server by using the K2P model. All values were set to default, except the value of relative gap width, which was set to 1, while the default gap width of 1.5 resulted in a single group. The bPTP method was performed on the web server at http://species.h-its.org, while the mPTP method was run on the web server at http://mptp.h-its.org/. Both methods were applied using default parameters, outgroups have been removed from the analysis and the same ML input tree was used.

## ﻿Results

### ﻿Taxonomic part

#### New species description

##### ﻿ Isoperla popijaci

Taxon classificationAnimaliaPlecopteraPerlodidae

Hlebec & Sivec sp. nov.

0F8F348F-51F1-5E07-A64B-2325D035AABF

http://zoobank.org/60B76C3E-14C2-4D5D-9587-C1931C87952B

**Figure 1.**
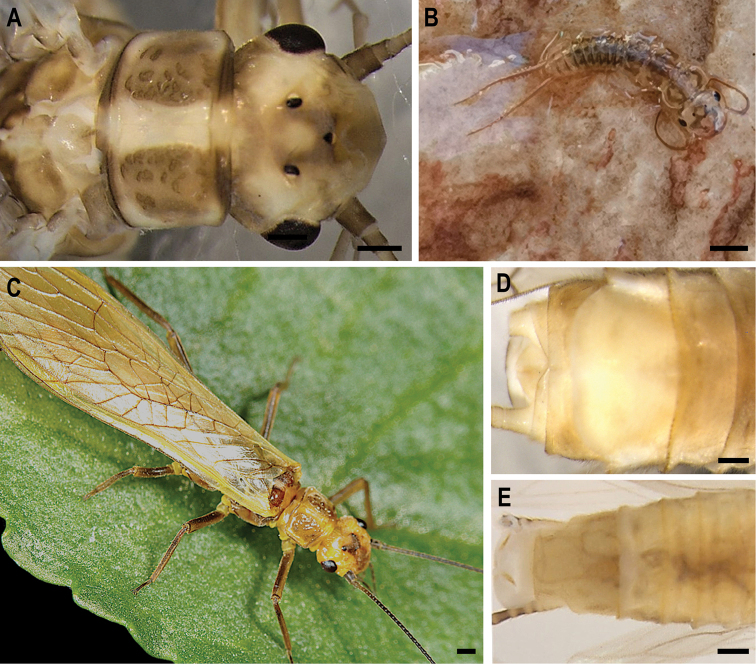
Morphology of *Isoperlapopijaci* sp. nov. **A** head and pronotum in dorsal view (adult male HT) **B** habitus (larval PT) **C** habitus (adult male PT) **D** female terminalia in ventral view (PT) **E** everted male copulatory organ (HT). Scale bar: 0.5 mm **A–E**.

**Figure 2.**
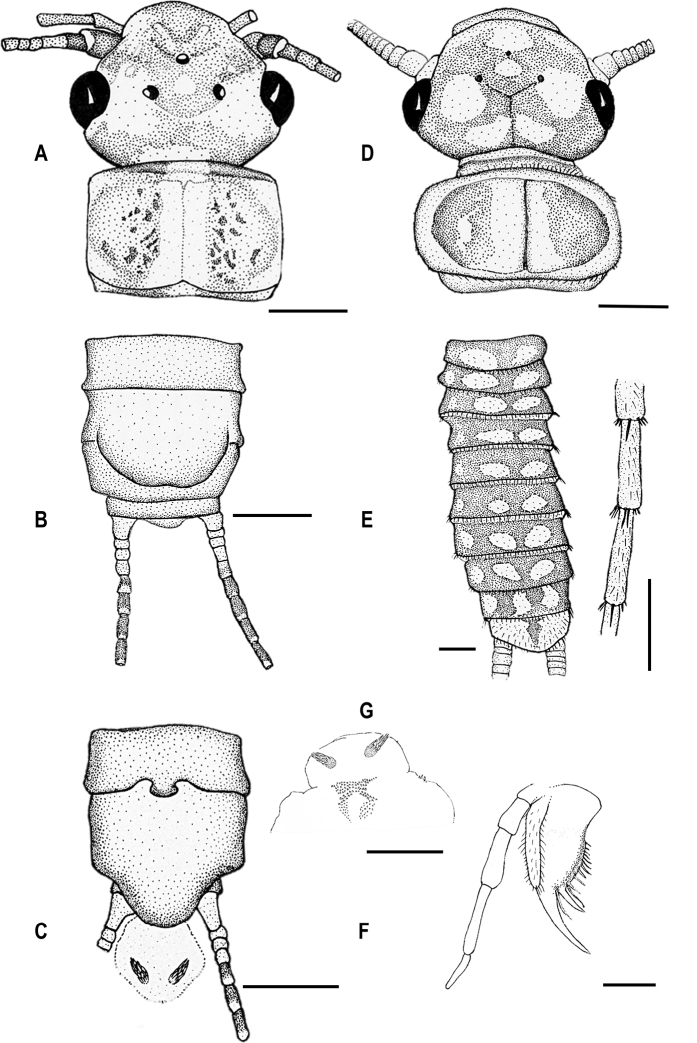
Morphology of *Isoperlapopijaci* sp. nov. **A** head and pronotum in dorsal view (adult female PT) **B** terminalia in ventral view (adult female PT) **C** terminalia in ventral view (adult male HT) **D** head and pronotum in dorsal view (larval PT) **E** abdomen in dorsal view and detail of a distal segment of a cercus (larval PT) **F** right maxilla in dorsal view (larval PT) **G** penial armature (adult male HT). Scale bars: 1 mm (**A–D**); 0.5 mm (**E–G**).

**Figure 3.**
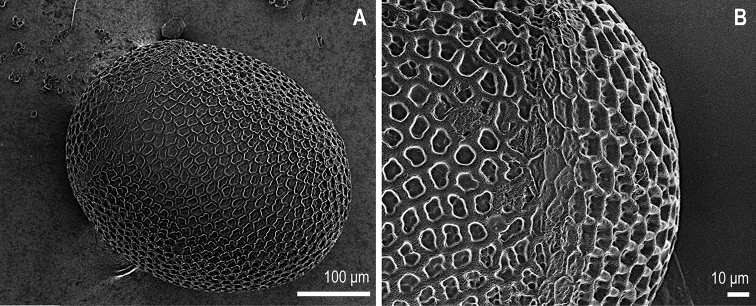
Egg of *Isoperlapopijaci* sp.nov. **A** whole egg, lateral view **B** detail of hatching line, lateral view

**Figure 4.**
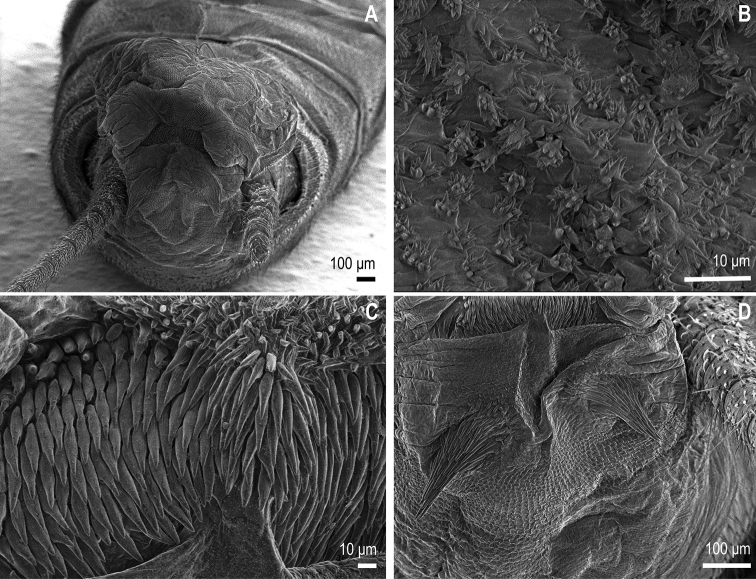
Extruded penis of *Isoperlapopijaci* sp. nov. **A** male abdomen with extruded penis, ventral view **B** detail of penial armature on the lateral lobe, dorsal view **C** scales of the upper medial penial armature, dorsal view **D** pair of the scales spike-like on the ventral lobe, dorsal view.

Figures 1A–E , 2A–G , 3A, B , 4A–D

###### Material examined

**(1♂ HT, 10♂♂ PTs, 23♀♀ PTs and 8 larvae PTs): 1♂HT** (96% ethanol) Original label: Croatia, Lika, Krbava field, Krasulja rivulet, karstic source Ševerova Cave; 44°40.78'N, 15°37.87'E, 21 June 2019, I. Sivec leg. (CNHM: CPSH); 6♂♂ **PTs** and 11♀♀ **PTs** (96% ethanol) same data as for the holotype; 5 larvae **PTs** (96% ethanol) 09 April 2015, I. Sivec leg.; 3 larvae **PTs** (96% ethanol) 22 February 2021, D. Hlebec leg.; 1♂**PT** and 3♀♀ **PT**s (96% ethanol) 2 June 2021, I. Sivec leg.; 3♀♀ **PTs** (96% ethanol) 18 June 2021, D. Hlebec leg.; 3♂♂ **PTs** and 6♀♀ **PTs** (96% ethanol) karstic source nearby village Mirići, 44°43.14'N, 15°38.09'E, 2 June 2021, I. Sivec leg.

###### Type material depository.

HT (1♂) and 31 PTs (7♂♂+18♀♀+6 larvae) in Zagreb, Croatia (CNHM), Collection of Plecoptera Sivec & Hlebec, under accession number CPSH 1–32; and 10 PTs (3♂♂+5♀♀+2 larvae) in Ljubljana, Slovenia (PMSL).

###### Type locality.

Croatia, Lika, Krbava field, Krasulja rivulet, karstic source Ševerova Cave, 44°40.78'N; 15°37.87'E; 640 m a.s.l.

###### Diagnosis.

The new species *I.popijaci* sp. nov. belongs to the *I.tripartita* species group, with divided medial penial armature into upper and lower coloured portions. It has, however, a specific penial armature on the ventral lobe of the penis, different from all known *Isoperla* species. The upper medial armature is subdivided, and the lower medial armature is present in two scale spike-like areas. The proximal part has a pair of drop-shaped areas armoured with spines, longer at the tip and shorter at the base. The medial penial armature with a field of shorter spines as in Figure 4C. Only a few irregular spines on the lateral side of the penis in the area of the upper armature of the penis.

###### Description.

Macropterous in both sexes, medium-sized species with yellow head and pronotum.

***Adult.*** Body length: **HT** male 18.5 mm; **PTs**: males 17–19 mm (n = 10), females 16.5–18 mm (n = 23).

Forewing length: **HT** male 12 mm; **PTs**: males 11–13.5 mm, females 11.5–14 mm.

***Colouration.*** General colour uniformly brownish (Figure 1C), slightly paler ventrally and laterally.

***Head.*** The central part of the head pale yellowish; darker at the lower part and between ocelli; slightly darker in the frontal and lateral part. M-line and tentorial callosities weakly expressed and inconspicuous. Pale spot positioned centrally between the ocelli, paler in the central distal part of the head. Eyes slightly smaller than the area delimited by the three ocelli. Scape and pedicel dark brown. Palpi uniformly cream coloured. The distal part of the antennae pale and the proximal segments darker (Figures 1A, 2A).

***Wings.*** Wings translucent brownish, venation dark brown.

***Pronotum.*** Pronotum yellowish, rectangular with angled edges. Medial and lateral parts of the pronotum pale; central part on both sides slightly darker and with dark brown textured surface (Figures 1A, 2A).

***Mesothorax and metathorax.*** Ventral surface of thorax uniformly brownish; dorsal side slightly darker, lateral part lighter. Mesonotum and metanotum predominantly dark brown.

***Legs.*** Femora and tibia brownish, same as body colouration. Tarsi slightly darker than femora and tibia on the dorsal side and pale ventrally.

***Male abdomen.*** Mesobasisternum and metabasisternum brown in the middle and darker laterally. Ventral surface of male abdomen uniformly brownish, slightly darker dorsally. A few proximal segments of cerci pale, with rest dark brown.

***Penis (everted).*** Divided into four lobes, with a basal section in everted position. The medial penial armature on the ventral surface of the penis divided into an upper and a lower part, both are coloured (Figures 2C, G, 4A), upper part rather pale. The upper medial penial armature is further subdivided into left and right arms, elongated, delimited from scales of the lateral lobes (Figure 4C). Length of the arms is 200–250 µm, width 100–120 µm. Scales of the upper medial penial armature forming a drop-shaped area, spike-like, with longer scales at the tip and shorter ones at the base. Length of the scales 25–37 µm, width 7–9 µm at the base. The lower part of the medial penial armature subdivided, with an irregular upturned V-shaped area and bearing very short spines (Figure 4D). Length of the areas 220–250 µm, width 100–140 µm. The scales are spike-like, thinner than in the upper medial armature. The ventral lobe hemispherical, covered with hair-like scales, in some places ciliated scales. The medial lobe small with diverse scales. Lateral penial armatures located on the lateral lobes, above the basal section, small and indistinct with only a few spines. Detail of the lateral lobe as in Figure 4B.

***Female abdomen.*** All tergites uniformly brownish. Sternites slightly paler brownish. A few basal segments of cerci pale, rest of cerci dark brown. Subgenital plate large and wide reaching near the end of sternite IX (widely concave in the middle) (Figure 2B).

***Egg.*** Chorion light brown, 0.34–0.38 mm long and 0.29–0.33 mm wide (n = 22). Chorion with marked ornamentation of irregular round shape. Follicular cell impressions with finer inner punctations. Hatching line distinct. Micropyles not well recognisable. Collar and anchor missing (Figure 3A, B).

***Larva.*** Body length of not-completely-mature larva 14–16 mm (n = 8). General colour pale brownish; with darker markings on head and abdomen. Body and legs typically pilose. Swimming hairs present on femora, tibiae and tarsi. Posterior abdominal fringe short and cercal fringe no longer than width of cercal segment. General colour of the head brownish, with a darker transversal mask connecting eyes and ocelli (Figure 2D). M line indistinct. Eyes well developed. Mouth parts and basal parts of antennae pale coloured; distal part of antennae dark brownish. Lacinia bidentate; inner margin with 4–5 stout setae and a row of short thin setae below subapical tooth. Pronotum rounded; brownish; with indistinct darker pattern centrally and distinctly paler laterally (Figure 2D). Pronotal setal fringe with short bristles and bearing only a few longer setae at posterior margin. Ventral side of the body and leg pale coloured. Abdominal tergites darker, brown with a pair of relatively large drop-shaped pale spots in the middle of the abdomen (Figure 2E). Paraprocts and cerci uniformly pale. Setation on distal section of cercal segments with rather uniform setae and single larger dorsal setae.

###### Etymology.

The specific name is the genitive singular of the Latinised version of the surname Popijač (Popiacus, -i, m.), given in honour of colleague Dr Aleksandar Popijač and his achievements in field research and knowledge of the Plecoptera fauna in Croatia.

###### Distribution and ecology.

**Figure 5.**
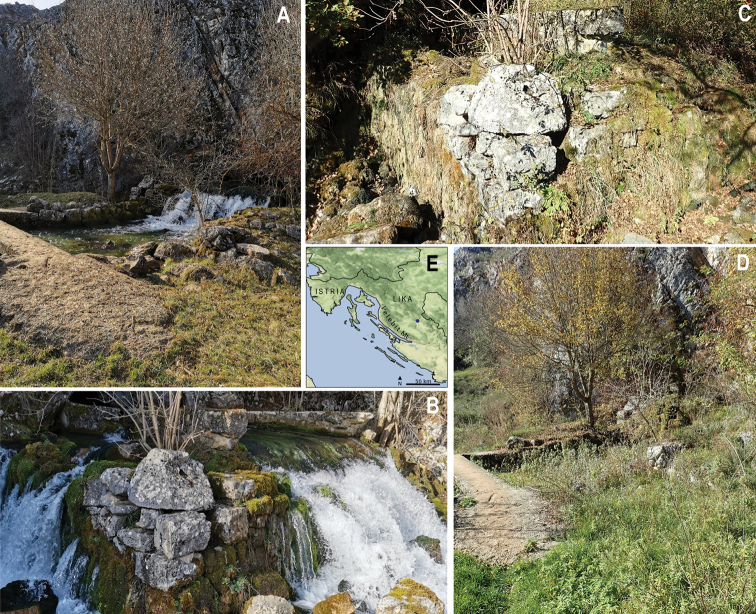
Type locality of the Popijač’s Yellow Sally, *Isoperlapopijaci* sp. nov.: Ševerova Cave in Croatia **A** and **B** photographs in wet phase **C, D** photographs in dry phase **E** map (blue circle indicates type locality).

The species was collected at the entrance to the Ševerova Cave, occasional karstic source of the intermittent Krasulja rivulet in Krbava field and two year later (on 2 June 2021) near the karstic source of the same rivulet, near the village of Mirići. The Ševerova Cave (old name Hrnjakova Cave) is located on the northern edge of the Krbava field (karst field located near settlement Krbavica in the vicinity of the Plitvice Lakes National Park). The temporary Krasulja rivulet is part of the hydrogeological system of the Krbavica River (Figure 5E). For several months a year, the water runs from the cave and forms the Krasulja rivulet, which flows into the Krbavica River and sinks on the south side of the field. When the discharge of the Krasulja falls below 60 l/sec, the water-flow ceases from Ševerova Cave (Malinar and Čepelak 2009). The stream does not have a rich stonefly fauna and the species found at this locality, except the newly-described species of *Isoperla*, are *Amphinemurastandfussi* (Ris, 1902) and *Nemouracinerea* (Retzius, 1783). The substrate at the collection site of larvae was mainly composed of larger fractions.

### ﻿Conservation status

The new species should probably be regarded as Critically Endangered (CR) or Vulnerable (VU) by the IUCN Criteria. Up to now, it is known only from the areas nearby two karstic sources.

### ﻿Phylogenetic part

**Figure 6.**
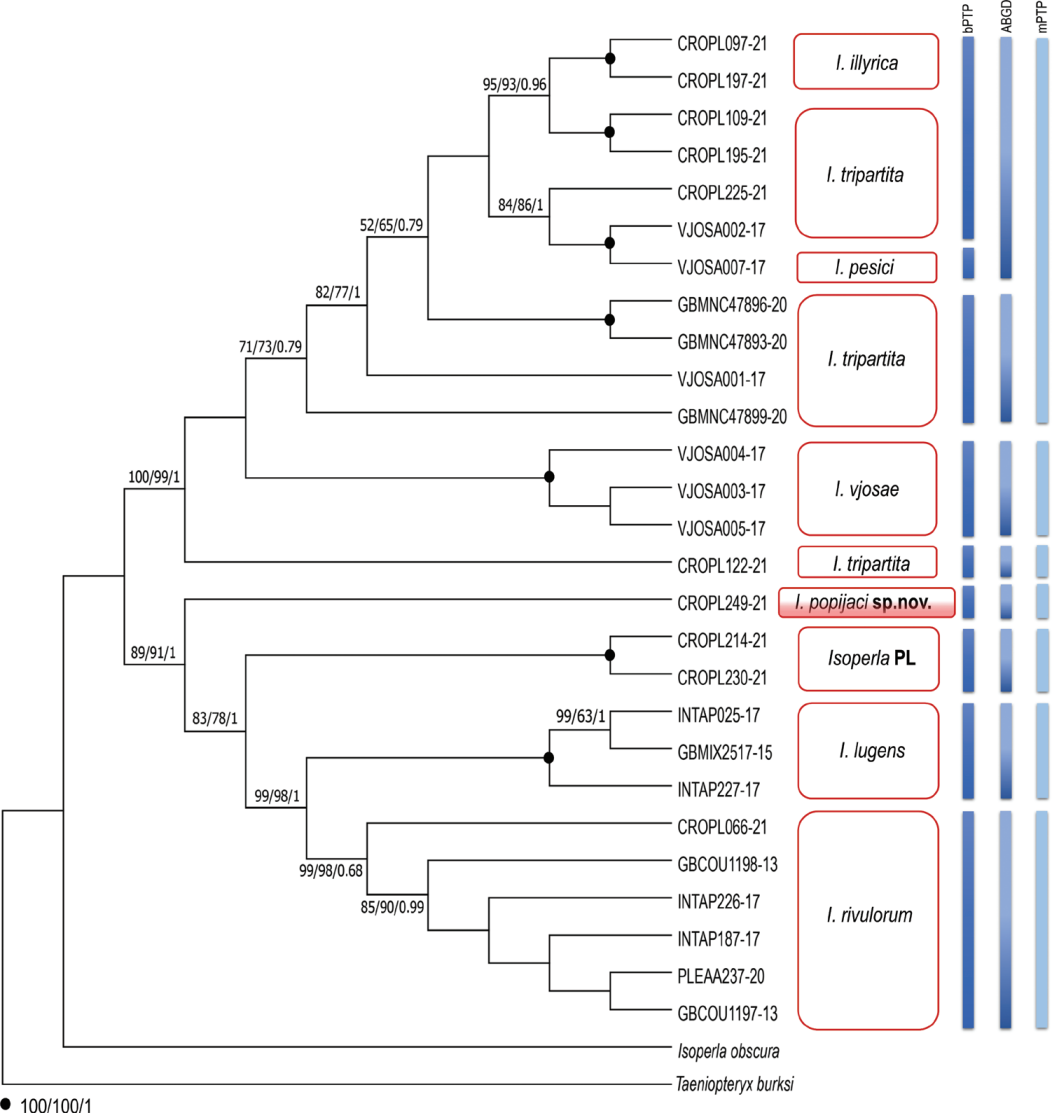
Maximum Likelihood cladogram, based on the analysis of the *COI* haplotypes of *Isoperla* species. Numbers at the nodes indicate Neighbour-Joining (NJ), Maximum Likelihood (ML) bootstrap support values (BS) and Bayesian posterior probabilities (BPP), respectively. The results of species delimitations are represented with the vertical bars, from left to right, indicate the OTUs inferred by bPTP, ABGD and mPTP. “*Isoperla* PL” indicates additional separate lineage obtained in this study. Terminal codes present BOLD/GenBank Process ID, as in Table 1.

The alignment of *COI* gene sequences was 658 bp in length and comprised of 202 variable sites, of which 139 were parsimony informative. Three implemented criteria of phylogenetic reconstruction (NJ, ML and BI) resulted in congruent topologies with highly similar support values (Figure 6), characterised by the presence of two deeply divergent lineages, *I.popijaci* sp. nov. and “*Isoperla* PL”, which did not cluster with any of the currently defined taxa.

Mitochondrial *COI* sequences, obtained from *I.popijaci* sp. nov. (adults and larva), were identical (a single unique haplotype). The monophyly of the newly-described species is highly supported (Figure 6). This species represents the first branch-off within the clade comprised of monophyletic *I.lugens* and *I.rivulorum* subclades, as well as another tentative new taxon obtained in this study (clade designated as “*Isoperla* PL” with representatives CROPL214–21 and CROPL230–21). The designation “PL” denotes the abbreviation Plitvice Lakes, nearby where a specimen was found. Five sequences of “*Isoperla* PL” represent 2 haplotypes (CROPL214–21 and CROPL230–21) with low intraspecific uncorrected *p*-distance (0.0096).

**Figure 7.**
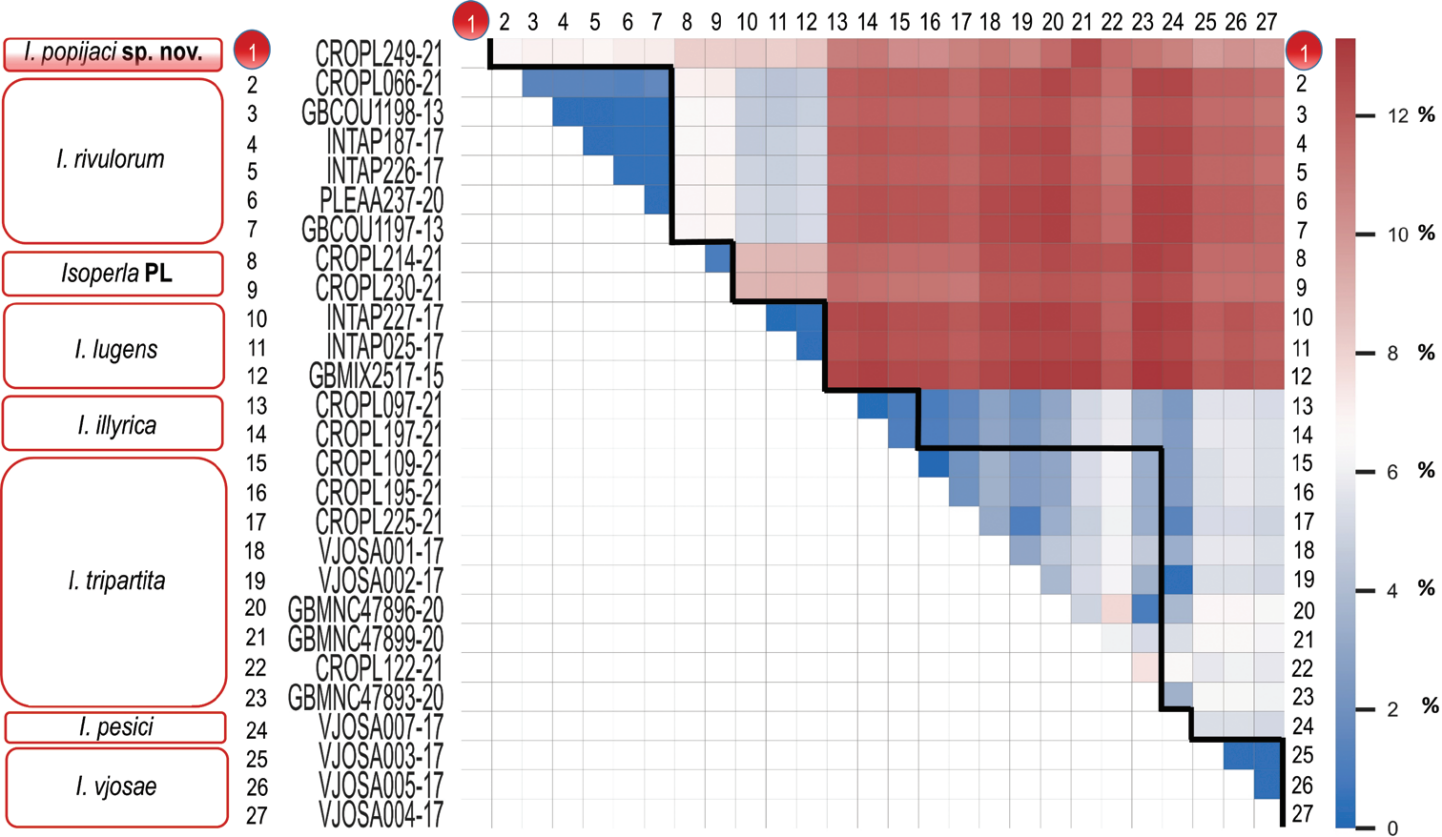
Colour heat map showing inter- and intraspecific uncorrected *p*-distances of the mitochondrial cytochrome oxidase subunit I (*COI*) barcode region. *Isoperlapopijaci* sp. nov. and *Isoperla* PL appear as highly divergent. Intraspecific *p*-distances are outlined by the black line.

Intraspecific uncorrected *p*-distances are as follows for the following species: 0.32–1.59% in *I.rivulorum*, 0.16–0.48% in *I.lugens*, 0.01–7.82% in *I.tripartita*, 0.32% in *I.vjosae* and 0.16% in *I.illyrica*. Interspecific uncorrected *p*-distances for *I.popijaci* sp. nov. ranged from 6.69–12.59%; specifically, 6.69–7.17% to *I.rivulorum*, 8.15–8.45% to *I.lugens*, 9.99–10.22% to *I.vjosae*, 10.4–12.6% to *I.tripartita*, 10.38–12.61% to *I.illyrica*, 10.69% to *I.pesici* and 8.12% to the “*Isoperla* PL” (Figure 7). Overall, observed intraspecific genetic distances within the genus ranged from 0.01–7.82%.

Within the *I.rivulorum* clade, Croatian sample CROPL066–21 appeared as a separate lineage, subdivided from Alpine specimens (Figure 6). The uncorrected *p*-distances for sample CROPL066–21 are in the range 1.28–1.59% to other *I.rivulorum* samples.

A well-supported clade comprised two newly-discovered lineages (*Isoperlapopijaci* sp. nov. and “*Isoperla* PL”), together with *I.lugens* and *I.rivulorum*, and was recovered in all three tree-building algorithms.

According to the results of the first molecular characterisation of *I.illyrica* obtained in this study, specimens clustered in a within the monophyletic clade with intraspecific uncorrected *p*-distances of 0.16%. Interspecific *p*-distances between *I.illyrica* and *I.tripartita* ranged from 0.96–5.91%.

All species delimitation analyses (bPTP, ABGD and mPTP) for mtDNA (*COI*) have delineated two well-separated lineages *Isoperlapopijaci* sp. nov. and “*Isoperla* PL” as tentative species. Applied methods resulted in various numbers of delineated groups. In the ABGD analysis, initial partitioning identified eight, while recursive partitioning showed the existence of nine putative species for the majority of prior intraspecific divergence values (P). The mPTP method delimited seven operational taxonomic units (OTUs) and, according to these results, is the most conservative approach, while the bPTP recognised 9 OTUs.

Contrary to ABGD and bPTP, the mPTP analysis shows *I.illyrica*, *I.tripartita* and *I.pesici*, morphologically assigned to *I. (tripartita)* species group, as a single OTU. These species are completely separated into three OTUs in the bPTP analysis. The separation of sample CROPL122-21 as a distinct species (*I.tripartita*) was supported by all three species delimitation methods.

## ﻿Discussion

### ﻿Phylogeny and genetic diversity

The lowest interspecific *p*-distance between *I.popijaci* sp. nov. and *I.rivulorum* was found to be 6.69%, indicating distinct species. This exceeds intraspecific divergences (ISD ≥ 2%) commonly used as one of the criteria for a delimitation of closely-related species in aquatic insects: Ephemeroptera, Plecoptera and Trichoptera (Ball et al. 2009; Zhou et al. 2009). Values above 2% have already been reported amongst Plecoptera (Zhou et al. 2010, Gill et al. 2015), which was probably caused by poor mobility of some Plecoptera species (Boumans and Baumann 2012) and, consequently, geographical isolation among populations.

The finding of the second well-separated lineage (“*Isoperla* PL”), most closely related to species *I.rivulorum* (interspecific *p*-distance from 6.54–7.19%) implies existence of another new species of the genus *Isoperla* (unpublished data). Taxa obtained in this study (*Isoperlapopijaci* sp. nov. and “*Isoperla* PL”) are separated by a large interspecific *p*-distance of 8.12%. Future research will seek to determine whether this value has repercussions to the geographical isolation and specificity of the (micro-) habitats in which the taxa were found.

Based on the occurrence of *I.lugens* (alpine species) and *I.rivulorum* (alpine, central European species) in the Dinaric karst and their appearance as the most recently diverged lineages within *I.popijaci* + “*Isoperla* PL” + *I.lugens* + *I.rivulorum* clade (Figure 6), it can be assumed that the Dinaric karst might represent the area of origin of those alpine species as well as the diversification centre from where they spread northwards. However, to test this hypothesis, data across the whole distributional range and use of other molecular markers (mitochondrial and nuclear as well) are necessary.

To establish a final phylogenetic relationship in the monophyletic *I.tripartita* species group, it is necessary to collect specimens from its entire range and use a multi-gene molecular approach as well.

Previous research showed the wide range of variability in intraspecific divergence within the order Plecoptera (Zhou et al. 2009; Gill et al. 2015; Stark et al. 2015) and uncorrected intraspecific *p*-distances from our study (0.01–7.82%) are consistent with the previously reported values.

### ﻿Systematic implications

Based on the morphological characteristics, the new species can be assigned to the *Isoperlatripartita* species group. The *I.tripartita* species group is characterised by the divided medial penial armature (into upper and lower coloured portions, divided or subdivided) and lateral penial armatures (Illies 1954; Murányi 2011; Murányi et al. 2016). Popijač’s Yellow Sally is characterised by divided medial penial armature, with the distal part bearing short spines, but with indistinct lateral penial armature. The genetic distinction, in combination with morphological features, is significantly different from all other species and promotes *I.popijaci* sp. nov. as a new species.

Phylogenetic reconstructions support the monophyly of the *I.tripartita* species group, which is, together with *I.grammatica*, notable by the high morphological variability of certain species (Zwick 1978; Murányi 2011). In Croatia, significant morphological variability has been also observed in *I.inermis* from different localities (personal observation), of which some are very similar to *I.difformis* (Central European species) in the penial armature. Therefore, future studies should investigate relationships between and within *Isoperla* populations from the Balkan Peninsula (e.g. Cetina River, National Park Plitvice Lakes, Kupa River and nearby springs in Slovenia) by applying a multi-gene approach.

Other species are somewhat less variable and occupy smaller distributional areas (as recently described species from Europe and Asia). Those endemics are of special interest to our study because it is assumed that more endemics species are likely to be discovered, especially in poorly-explored areas with high biodiversity like the Balkans. More new species are expected to be found in Croatia, as the majority of the country’s territory has not been studied yet regarding Plecoptera.

Anthropogenic activities have already resulted in the reduction of population size (especially larger species from the genera *Perla*, *Dinocras* and *Perlodes*) (personal observation). All the above-mentioned calls for more detailed studies of species distributional patterns, as well as of genetic diversity of populations. Emphasis should also be put on the isolated habitats (karst areas) as they can have the highest conservation value as refugium and the maintenance of genetic diversity.

### ﻿Cave-dependent stoneflies?

Until now, Popijač’s Yellow Sally is known to inhabit the parts of the rivulet close to two karstic sources, of which one is a cave entrance. Although there are no true troglobionts within the order Plecoptera, several species have been found to inhabit stream sources around the openings of caves (for example *I.inermis*) and there are no records of these species from the downstream part of the same stream. Another example is *Brachypteratristis* (Klapálek, 1901), a species that spends its entire life cycle underground (the stream of Krupa River) (personal observations). It is, hence, important to pay special attention to the research of caves, pits, underground and temporary rivers and streams that abound in the Dinaric karst geology. These habitats host some of the most complex and diverse faunas (Culver and Sket 2000) as a consequence of composite geological history and the intensive process of karstification (Sket 1999). The Balkan Peninsula is known for its high biodiversity (Sket et al. 2004), especially of aquatic species (Kryštufek et al. 2007, Previšić et al. 2009, 2014; Murányi 2011; Vitecek et al. 2015; Kučinić et al. 2017). It can be expected that future research will contribute to the discovery of biodiversity patterns as well as new species, especially microendemic species (Graf et al. 2009, 2012; Kučinić et al. 2013; Vitecek et al. 2017). Karst habitats, such as Ševerova Cave, represent some of the most dynamic freshwater habitats, especially in terms of biological-geological interactions (Ridl et al. 2018). With the alternation of wet and dry phases and temporal dynamics of water flow, temporary rivers have a great influence on local ecological interactions, both in aquatic and terrestrial habitats (Larned et al. 2010). It is a significant assumption that climate change will increase the duration and frequency of dry phases, so it is expected that this will lead to the disappearance of taxa whose entire life cycle (or at least part of it) is related to aquatic environments (Larned et al. 2010).

## ﻿Conclusions

*Isoperlapopijaci* sp. nov. is probably a stenoendemic Yellow Sally species found at two karstic sources of the intermittent Krasulja rivulet in Lika (Croatia), which has morphological characteristics similar to species from the *I.tripartita* species group. Phylogenetic analysis revealed the well-supported sister-group relationship of *I.lugens* and *I.rivulorum* and a basal position of *I.popijaci* sp. nov. relative to this clade. Considering its restricted distribution, *Isoperlapopijaci* sp. nov. should have the highest priority in conservation efforts.

## Supplementary Material

XML Treatment for Isoperla popijaci
